# Blood Pressure After Endovascular Thrombectomy and Malignant Cerebral Edema in Large Vessel Occlusion Stroke

**DOI:** 10.3389/fneur.2021.707275

**Published:** 2021-10-20

**Authors:** Xianjun Huang, Junfeng Xu, Ke Yang, Youqing Xu, Lili Yuan, Qiankun Cai, Xiangjun Xu, Qian Yang, Zhiming Zhou, Shuanggen Zhu, Xinfeng Liu

**Affiliations:** ^1^Department of Neurology, The First Affiliated Hospital of Wannan Medical College, Wuhu, China; ^2^Department of Neurology, The Second Affiliated Hospital of Fujian Medical University, Quanzhou, China; ^3^Department of Neurology, The Affiliated Central Hospital of Shenzhen Longhua District, Guangdong Medical University, Shenzhen, China; ^4^Department of Neurology, People's Hospital of Longhua, Shenzhen, China; ^5^Department of Neurology, Jinling Hospital, Medical School of Nanjing University, Nanjing, China; ^6^Division of Life Sciences and Medicine, Stroke Center and Department of Neurology, The First Affiliated Hospital of USTC, University of Science and Technology of China, Hefei, China

**Keywords:** stroke, thrombectomy, cerebral edema, blood pressure, large vessel occlusion

## Abstract

**Background:** Elevated blood pressure (BP) can cause blood–brain barrier disruption and facilitates brain edema formation. We aimed to investigate the association of BP level after thrombectomy with the development of malignant cerebral edema (MCE) in patients treated with endovascular thrombectomy (EVT).

**Methods:** Consecutive patients who underwent EVT for an anterior circulation ischemic stroke were enrolled from three comprehensive stroke centers. BP was measured hourly during the first 24 h after thrombectomy. MCE was defined as swelling causing a midline shift on the follow-up imaging within 5 days after EVT. Associations of various BP parameters, including mean BP, maximum BP (BP_max_), and BP variability (BPV), with the development of MCE were analyzed.

**Results:** Of the 498 patients (mean age 66.9 ± 11.7 years, male 58.2%), 97 (19.5%) patients developed MCE. Elevated mean systolic BP (SBP) (OR, 1.035; 95% CI, 1.006–1.065; *P* = 0.017) was associated with a higher likelihood of MCE. The best SBP_max_ threshold that predicted the development of MCE was 165 mmHg. Additionally, increases in BPV, as evaluated by SBP standard deviation (OR, 1.061; 95% CI, 1.003–1.123; *P* = 0.039), were associated with higher likelihood of MCE.

**Interpretation:** Elevated mean SBP and BPV were associated with a higher likelihood of MCE. Having a SBP_max_ > 165 mm Hg was the best threshold to discriminate the development of MCE. These results suggest that continuous BP monitoring after EVT could be used as a non-invasive predictor for clinical deterioration due to MCE. Randomized clinical studies are warranted to address BP goal after thrombectomy.

## Introduction

Malignant cerebral edema (MCE) is one of the serious clinical events in large-vessel occlusion stroke (LVOS), as it can lead to rapid neurologic deterioration (1). In recent years, endovascular thrombectomy (EVT) has been confirmed to improve functional outcomes for selected patients with LVOS of the anterior circulation (2). However, MCE is still a common phenomenon after thrombectomy (3). Moreover, the presence of MCE indicates a reduced likelihood of good functional outcomes and a higher likelihood of mortality (3, 4). Therefore, it is important to identify factors that can predict MCE in patients who have undergone EVT. A meta-analysis showed that younger age, a higher National Institutes of Health Stroke Scale (NIHSS) score, and large ischemic signs on computed tomography are reliable predictors for MCE (5). Nevertheless, these factors cannot be intervened.

Blood pressure (BP) management following treatment of LVOS patients with EVT is an important scientific question (6). In a recently published meta-analysis, an elevated systolic BP (SBP) level after EVT was associated with poor outcomes in patients with LVOS (7). Moreover, moderate BP control (<160/90 mmHg) seems to be associated with better clinical outcomes and lowers the odds of 3-month mortality (8, 9). Theoretically, elevated BP could cause blood–brain barrier (BBB) disruption and facilitate cerebral edema (CED) formation (10). Moreover, higher BP after reperfusion may exacerbate the reperfusion injury (11). These mechanisms may result in the development of MCE. However, data on the association of BP level after EVT with the development of MCE are relatively scarce.

Therefore, in this study, we investigated the association between mean BP level after thrombectomy and the development of MCE in patients treated with EVT. Moreover, determined the best post-EVT maximum BP (BP_max_) threshold that predicts the development of MCE. Finally, we evaluated the effect of BP variability (BPV) on the development of MCE.

## Methods

### Study Participants

This study was a retrospective analysis of a prospective registry. We enrolled anterior circulation LVOS patients who underwent EVT at three comprehensive stroke centers (Jinling Hospital between January 2014 and December 2018, Yijishan Hospital between July 2015 and December 2019 and the second affiliated Hospital of Fujian Medical University between January 2016 and December 2019). The study was approved by the local ethics committee.

**Figure 1:**
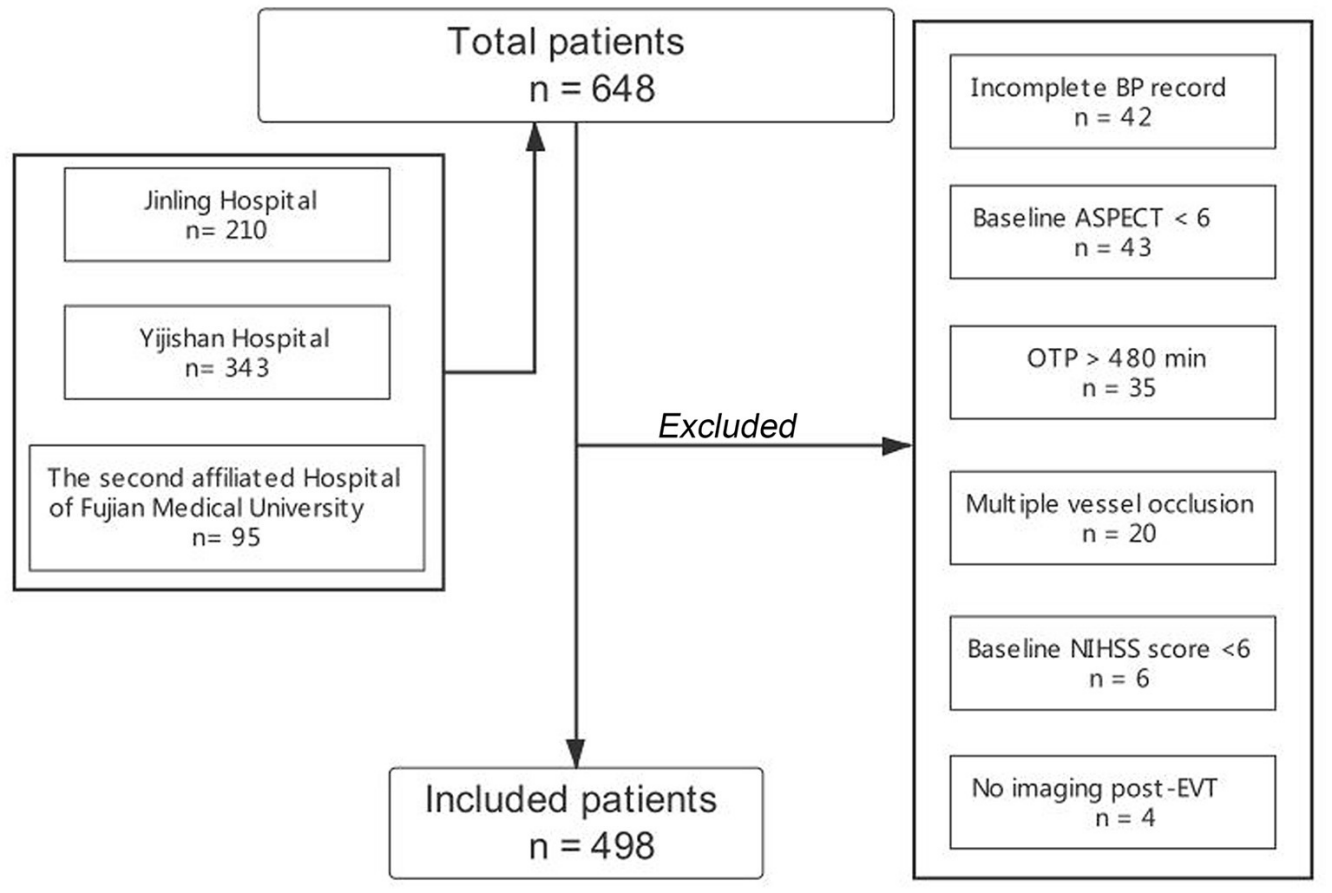
Flow chart of the study population.

Patients were included if they fulfilled the following inclusion criteria: (1) age ≥ 18 years; (2) onset to puncture time (OTP) ≤ 480 min; (3) admission NIHSS score ≥ 6, admission Alberta Stroke Program Early CT (ASPECT) score ≥ 6, and pre-stroke modified Rankin Scale (mRS) score <2; and (4) patients with LVOS, including the internal carotid artery (ICA), the middle cerebral artery (MCA) or the anterior cerebral artery (ACA) occlusion. We excluded patients with multiple vessel occlusion (MVO), incomplete BP record, and without post-procedural imaging. Additionally, EVT was performed under local anesthesia in all centers. The flow chart of the inclusion of the study population is displayed in Figure 1.

### Data Collection

All consecutive patients were prospectively documented. The clinical data included age, sex, medical history of hypertension, diabetes mellitus type 2, admission NIHSS and ASPECT scores, and the Trial of ORG 10172 in Acute Stroke Treatment (TOAST) classification.

BP data were consecutively recorded 24 h after EVT. BP goal was determined by the operator upon the end of thrombectomy or according to institutional protocol. BP was measured by non-invasive BP monitoring devices each hour during the first 24 h after EVT. Mean BP, BP_max_, and BPV during the first 24 h were analyzed. BP_max_ was defined as the highest BP value in 24 h after EVT.

We calculated BPV for both SBP and diastolic BP (DBP) using three statistical methodologies: standard deviation (*SD*), coefficient of variation (*CV*), and successive variation (*SV*). *SD* was defined as the dispersion of a dataset relative to its mean and is calculated as the square root of the variance by determining the variation between each data point relative to the mean. *CV* was defined as the ratio of the *SD* and the mean. *SV* was calculated as the square root of the average squared difference between two successive BP measurements.

In addition, data on the use of continuous intravenous antihypertensive agents were collected for analysis.

The procedural variables were recorded by the operators, including OTP, onset to reperfusion time (OTR), occlusion location, the first thrombectomy approach (stent retriever first, aspiration first, angioplasty, or stent first), bridging therapy, recanalization status, and collateral status.

Recanalization status was evaluated by the modified Thrombolysis in Cerebral Infarction (mTICI) grading system. Successful recanalization was defined as an mTICI score of 2b or 3. Collateral circulation was assessed according to retrograde contrast opacification of vessels within the occluded area on delayed pre-treatment digital subtraction angiography (DSA) images (12). The collateral grading was classified as follows: Grade 0 was assigned if there was no significant reconstitution in the territory of the occluded vessel or if the collateral flow reached less than one-third of the occluded vessel territory, grade 1 was assigned if the collateral flow reached less than two-thirds and more than one-third of the occluded vessel territory, and grade 2 was assigned if the collateral flow reached more than two-thirds of the occluded vessel territory or the proximal main stem. Good collateral circulation was defined as grade 2, and poor collateral circulation was defined as grade 0–1.

### Definition of Malignant Cerebral Edema

For all included patients, the imaging characteristics were assessed by two experienced neurologists/interventionists (ZM Zhou and Q Yang), who were blinded to the clinical data. In the event of discrepancies, the final result was determined by consensus opinion. Referring to previous studies (13), CED subtypes were defined as follows: CED-1 was defined as brain swelling comprising less than one-third of the hemisphere, CED-2 was defined as swelling comprising more than one-third of the hemisphere, and CED-3 was defined as swelling causing midline shift. MCE were defined as CED-3. In these patients, CED was evaluated via the follow-up head computed tomography within 5 days after EVT.

### Statistical Analysis

Continuous variables are presented as the mean ± standard deviation (*SD*) or as the median (interquartile range, IQR). Categorical variables are presented as percentages. Continuous variables were analyzed using the Mann-Whitney U test. Categorical variables were analyzed using the chi-square test or Fisher's exact test as appropriate. The threshold of SBP_max_ that best discriminated the development of MCE was determined by the area under the receiver operating characteristic curve (ROC) and Youden index. Multivariate logistic regression models were computed for the prediction of odds of MCE. The variables with *P* < 0.1 from the univariate analysis were entered into the logistic regression. Regression coefficients and odds ratios (OR) with two-sided 95% confidence intervals (CIs) for each of the variables included in the model were finally calculated. Repeated BP measurements were analyzed using the generalized estimating equation (GEE) method. All statistical analyses were computed using SPSS 25 (IBM Corp., Armonk, NY, USA).

## Results

During the study period, 648 anterior circulation LVOS patients who underwent EVT were registered in the three centers. A total of 498 patients were enrolled in the final cohort for analysis after excluding 150 patients due to incomplete BP record (*n* = 42), admission ASPECT score <6 (*n* = 43), no imaging after EVT (*n* = 4), OTP > 480 min (*n* = 35), patients with MVO (*n* = 20), and admission NIHSS score <6 (*n* = 6).

Of 498 patients, the mean age was 66.9 ± 11.7 years and 290 (58.2%) were male. The median NIHSS and ASPECT scores on admission were 16 (IQR13-20) and 9 (IQR8-10), respectively. The mean OTP time was 262.4 ±79.8 min, and the mean OTR time was 353.3± 93.6 min. Among the included patients, 219 (44%) had 3-month mRS 0–2. The baseline characteristics of the patients are shown in Table 1.

**Table 1:** Demographics and baseline characteristics stratified by MCE.

	**All patients (*n =* 498)**	**MCE (*n =* 97)**	**Non-MCE (*n =* 401)**	***P***
Age, mean (SD), y	66.9 (11.7)	67 (10.9)	66.9 (11.8)	0.995
Male, *n* (%)	290 (58.2)	55 (56.7)	235 (58.6)	0.733
No. of BP measurements per patient, mean (SD)	22 (4.5)	22 (3.7)	21 (4.6)	0.878
**Medical history**, ***n*** **(%)**				
Hypertension	335 (67.3)	75 (77.3)	260 (64.8)	0.019
Diabetes mellitus	101 (20.3)	23 (23.7)	78 (19.5)	0.349
AF	236 (47.4)	47 (48.5)	189 (47.1)	0.815
**Clinical characteristics, median (IQR)**				
Baseline SBP, mmHg	145 (128–160)	150 (132–160)	143 (128–160)	0.085
Baseline DBP, mmHg	80 (72–91)	83 (70–95)	80 (74–90)	0.558
Admission NIHSS scores	16 (13–20)	18 (15–21)	15 (12–19)	<0.001
Admission ASPECT scores	9 (8–10)	8 (7–9)	9 (8–10)	<0.001
**TOAST classification**, ***n*** **(%)**				0.113
LAA	169 (33.9)	27 (27.8)	142 (35.4)	
Cardioembolic	277 (55.6)	58 (59.8)	219 (54.6)	
Undetermined or others	52 (10.5)	12 (12.4)	40 (10)	
**Occlusion location**, ***n*** **(%)**				0.001
ICA	209 (42)	64 (66)	145 (36.2)	
MCA/ACA (M1/A1)	254 (51)	28 (28.9)	226 (56.4)	
MCA/ACA (M2/A2)	35 (7)	5 (5.2)	30 (7.5)	
OTP, mean (SD), min	262.4 (79.8)	259.5 (76.1)	263.1 (80.8)	0.679
OTR, mean (SD), min	353.3 (93.6)	377.9 (90.8)	347.3 (93.4)	<0.001
**Collateral score**, ***n*** **(%)**				<0.001
Grade 0	92 (18.5)	37 (38.1)	55 (13.7)	
Grade 1	197 (39.6)	41 (42.3)	156 (38.9)	
Grade 2	209 (42)	19 (19.6)	190 (47.4)	
Bridging treatment, *n* (%)	119 (23.9)	30 (30.9)	89 (22.2)	0.070
**Type of procedure**, ***n*** **(%)**				0.194
Stent retriever first	389 (78.2)	79 (81.4)	310 (77.3)	
Aspiration first	59 (11.8)	13 (13.4)	46 (11.5)	
Angioplasty or stent first	50 (10)	5 (5.2)	45 (11.2)	
Continuous intravenous antihypertensive agents, *n* (%)	306 (61.4)	61 (62.9)	245 (61.1)	0.745
mTICI, 2b/3, *n* (%)	364 (73.1)	51 (52.6)	313 (78.1)	<0.001
90-day mRS 0–2, *n* (%)	219 (44)	8 (8.2)	211 (52.6)	<0.001

*ASPECT, Alberta Stroke Program Early CT; AF, atrial fibrillation; ACA, anterior cerebral artery; DBP, diastolic blood pressure; ICA, internal carotid artery; LAA, large-artery atherosclerosis; mTICI, modified Thrombolysis in Cerebral Infarction; MCA, middle cerebral artery; NIHSS, National Institutes of Health Stroke Scale; OTP, symptoms onset to groin puncture time; OTR, symptoms onset to reperfusion; SBP, systolic blood pressure*.

### Serial BP Measurements and MCE

Among the enrolled patients, 97 (19.5%) patients developed MCE. We did not find differences in baseline BP between the patients with MCE and without MCE. However, patients with MCE had significantly higher mean SBP (128 mmHg vs. 123 mmHg, *P* < 0.001) after EVT than those without MCE. Moreover, in the multivariate logistic regression models, increases in mean SBP (OR, 1.035; 95% CI, 1.006–1.065; *P* = 0.017) were associated with a higher likelihood of MCE.

**Figure 2:**
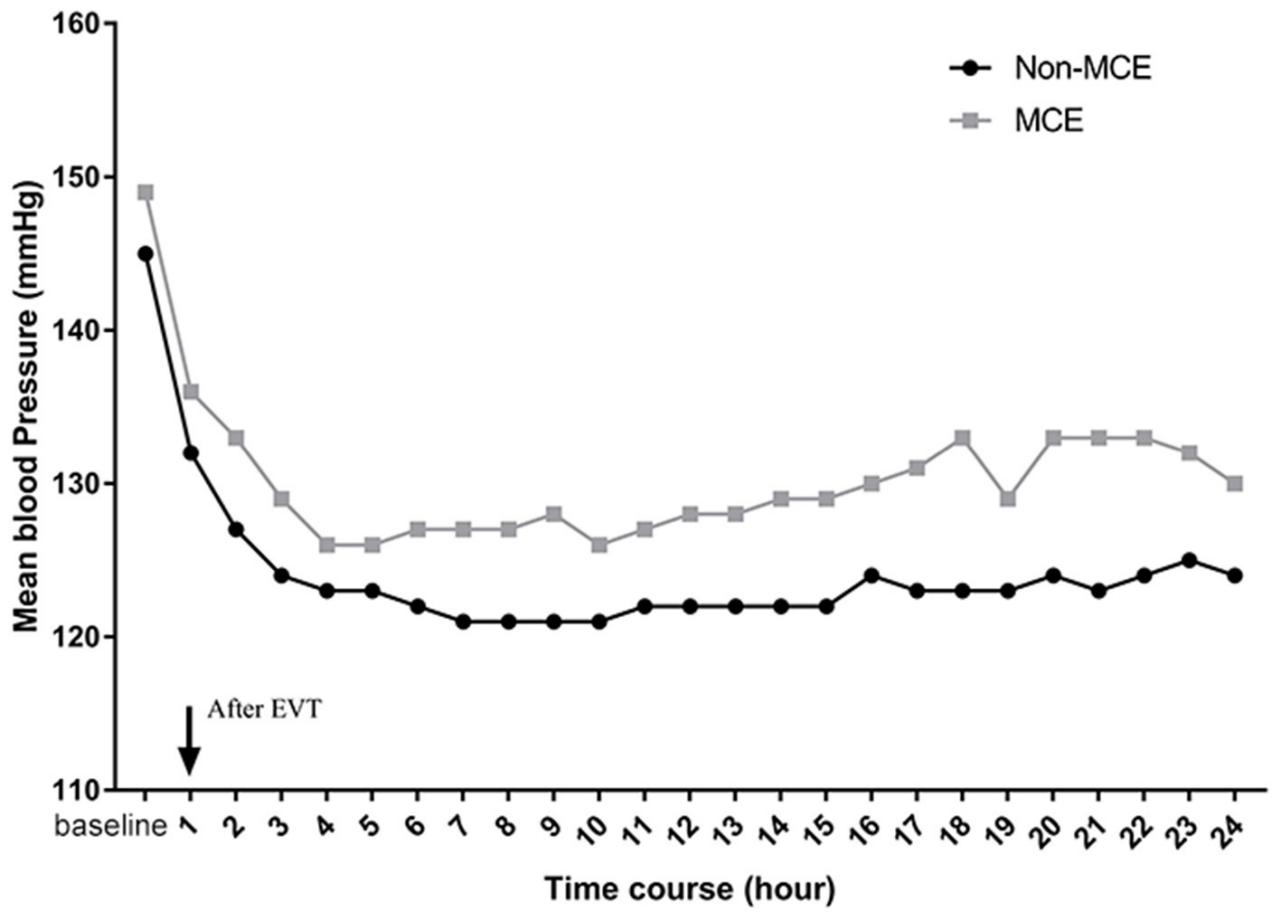
Serial SBP levels plotted according to the development of MCE.

Additionally, a significant association was observed between BP serial measurements after thrombectomy and MCE (Figure 2). In patients with MCE, SBP throughout the 24 h post-EVT was higher than that in patients without MCE (*P* = 0.036 by the GEE method for MCE).

### The Optimal Threshold of MCE-Predicting BP_max_ Value

**Figure 3:**
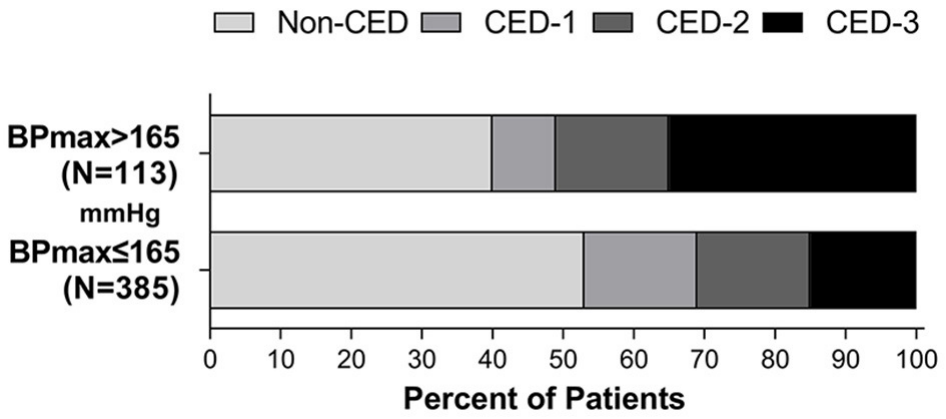
Distribution of CED in patients with a SBP_max_ of ≤ 165 mm Hg and >165 mmHg during the first 24 h after EVT.

After adjusting the admission NIHSS and ASPECT scores, the history of hypertension, baseline BP, OTR, occlusion location, bridging treatment, collateral circulation and mTICI, higher BP_max_ (OR, 1.021; 95% CI, 1.006–1.036; *P* = 0.007) were associated with a higher risk of developing MCE. The ROC-derived optimal cut-off value with Youden's index for predicting MCE was 165 mm Hg (41.2% sensitivity, 95% CI, 31.5–51.7%, 81.8% specificity, 95% CI, 77.6–85.4%; area under the curve 0.615; 95% CI, 0.549–0.681, *P* < 0.001). Binary logistic regression analysis showed that patients with SBP_max_ >165 mm Hg (OR, 2.729; 95% CI, 1.526–4.880; *P* = 0.001) was also associated with a higher likelihood of MCE. The distribution of CED in patients with SBP_max_ of ≤ 165 mm Hg and >165 mm Hg is shown in Figure 3.

**Figure 4:**
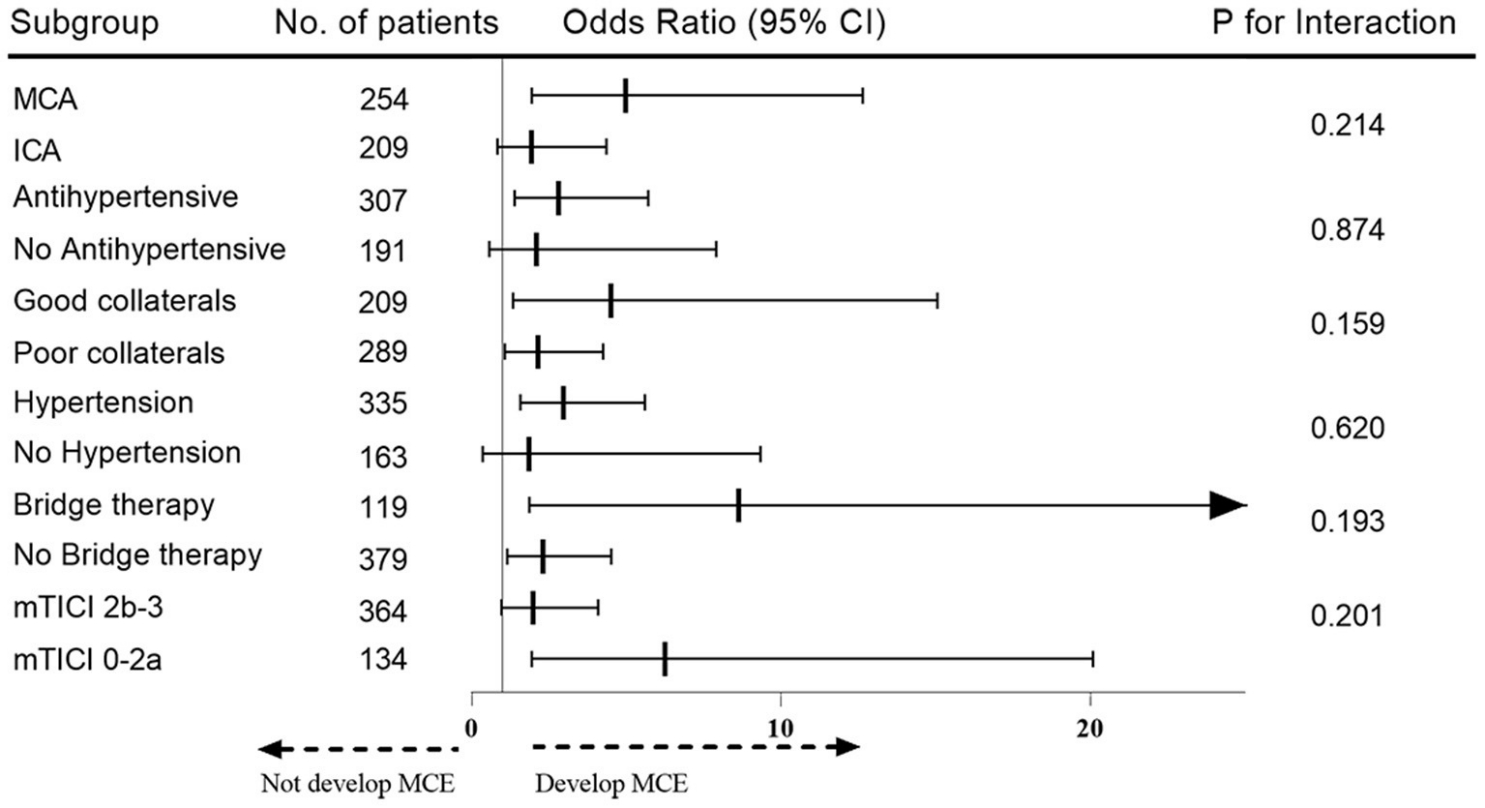
Subgroup analysis for heterogeneity of effect of SBP_max_ >165 mmHg on the development of MCE. The odds of developing MCE for each subgroup is depicted with a square and a line, representing a 95% CI on the forest plot on the right.

In subgroup analyses, no heterogeneity in the effect of SBP_max_ >165 mm Hg on the development of MCE was observed according to subgroups of patients based on history of hypertension, collateral status, continuous intravenous antihypertensive agents, bridge therapy, occlusion site and recanalization status after correction for multiplicity (Figure 4). However, the adjusted ORs were not significant for patients with successful recanalization (OR, 1.991; 95% CI, 0.970–4.086; *P* = 0.061), those without history of hypertension (OR, 1.861; 95% CI, 0.370–9.356; *P* = 0.451), those with ICA occlusion (OR, 1.920; 95% CI, 0.845–4.365; *P* = 0.119) and in those without intravenous antihypertensive agent (OR, 2.097; 95% CI, 0.557–7.892; *P* = 0.274).

### Blood Pressure Variability and MCE

We further evaluated the effect of BPV on the development of MCE. In the multivariate logistic regression models, increases in SBP_SD_ (OR, 1.061; 95% CI, 1.003–1.123; *P* = 0.039) were associated with higher likelihood of MCE. There was no association for SBP_CV_, SBP_SV_, or any DBP parameters with the outcome parameters in the multivariate logistic models. Associations of BP parameters with MCE are shown in Table 2.

**Table 2:** Association of blood pressure parameters with the development of MCE.

	**Unadjusted**			**Adjusted**		
	**MCE**	**Non-MCE**	***P***	**OR**	**95% CI**	***P***
**SBP**						
Mean	128 (120–136)	123 (116–131)	<0.001	1.035	1.006–1.065	0.017
Max	148 (139–161)	158 (144–175)	<0.001	1.021	1.006–1.036	0.007
CV	9.05 (7.19–11.73)	10.02 (8.39–14.30)	<0.001	1.065	0.981–1.018	0.948
SV	12.32 (9.66–15.64)	14.73 (11.68–18.99)	<0.001	1.031	0.989–1.075	0.149
SD	11.19 (8.81–14.23)	13.31 (10.36–18.71)	<0.001	1.061	1.003–1.123	0.039
**DBP**						
Mean	73 (66–79)	70 (65–76)	0.071	0.990	0.954–1.028	0.606
Max	89 (81–98)	95 (84–101)	0.001	1.012	0.989–1.036	0.313
CV	11.95 (9.56–14.87)	13.45 (11.17–15.89)	0.002	0.999	0.927–1.078	0.987
SV	9.56 (7.58–12.02)	10.66 (8.88–13.80)	0.001	1.015	0.939–1.098	0.708
SD	8.42 (6.61–10.62)	9.44 (8.08–11.45)	<0.001	1.009	0.907–1.122	0.872

*Adjusted for: baseline NIHSS and ASPECT scores, baseline BP, Hypertension, OTR, occlusion location, bridging treatment, collateral circulation and mTICI*.

*CV, coefficient of variation; DBP, diastolic blood pressure; max, maximum; SBP, systolic blood pressure; SD, standard deviation; SV, successive variation*.

## Discussion

In this multicenter observational study, the main findings were as follows: First, patients with a higher mean SBP during the first 24 h after EVT were more likely to develop MCE. Moreover, the serial SBP measurements demonstrated that patients with MCE had significantly higher SBP values than those without MCE. Second, the best SBP_max_ threshold that predicted the development of MCE was 165 mmHg. Furthermore, subgroup analyses also revealed similar direction and size for the effect of post-EVT SBP_max_ >165 mm Hg on the development of MCE. Third, BPV, as evaluated by SBP_SD_, was associated with the development of MCE in EVT-treated patients.

MCE is the leading cause of neurologic deterioration or death within the first days after ischemic stroke (1). Our previous study revealed that MCE is a common clinical condition in patients who underwent EVT and significantly impacts on clinical outcomes (4). Similar to the previous study, we found that MCE after EVT was not uncommon (19.5%).

BP management following EVT in LVOS patients is increasingly being taken seriously (6). Furthermore, BP is a readily modifiable parameter with the potential to improve outcomes. Recent studies have shown that elevated SBP level and variability after EVT are associated with poor outcomes among patients with LVOS (14, 15). Moreover, lowering of BP within the first 24 h after EVT may have a positive impact on clinical outcomes in treated patients (16). However, studies examining the association of BP parameters after procedure with MCE are largely lacking.

In the present study, we found that LVOS patients with a higher mean SBP during the first 24 h after EVT were more likely to develop MCE. This result is in accordance with previous studies (10, 17). Vemmos et al. reported that elevated SBP values in the acute period are associated with subsequent brain edema formation in ischemic and hemorrhagic stroke patients (10). Additionally, Serena et al. found that in patients with large MCA infarction, an increased risk of fatal brain edema is associated with history of hypertension (17). However, the pathophysiological mechanisms of higher BP leading to MCE are not fully understood.

A possible explanation of our findings is that elevated SBP during the first 24 h after EVT causes BBB disruption and facilitates brain edema formation (1). MCE is initially cytotoxic characterized by intracellular water accumulation and later vasogenic, in which water moves across the BBB into the extracellular interstitial space (18). An early experimental study indicated that elevated BP may facilitate edema formation by increasing BBB permeability (19). Additionally, the reperfusion injury may play an important role in the development of MCE after EVT (20–22). Yang and Betz found that BBB disruption was exacerbated after reperfusion and vasogenic edema was associated with increased the BBB disruption after reperfusion in a rodent model (21). Finally, in the setting of ischemic stroke, elevated BP could promote a deleterious proinflammatory state (11), which may result in the development of brain edema. However, the mechanism needs further study.

Another interesting finding of this study was the pattern of SBP trends in the 24 h post-EVT. After EVT, there was a decline in SBP. The results were similar to those of recent studies. For example, Cho et al. found that SBP decreased steeply during the first 5–7 h after EVT and then achieved a plateau for 24 h (23). A multicenter prospective cohort study (Blood Pressure after Endovascular Therapy for Ischemic Stroke, BEST) also demonstrated a decline in BP in patients with LVOS after EVT (15). Additionally, our study further indicated that, in patients with MCE, SBP throughout the 24 h post-EVT was higher than that in patients without MCE. Although iatrogenic BP lowing after EVT may affect the understanding of this finding, we did not find any significant difference in the use of intravenous antihypertensive drugs between the two groups. In addition, in our study, the best SBP_max_ threshold that predicted the development of MCE was 165 mmHg. BEST study also showed that a peak SBP around 160 mm Hg in the 24 h post-EVT best dichotomizes good vs. bad functional outcomes (15). Moreover, a recent large sample study demonstrated that post-thrombectomy SBP <160 mmHg following successful revascularization with EVT seem to be related with better clinical outcomes than SBP <180 mmHg (9). Thus, the current study further expanded our understanding of the association of BP control with outcomes in patients with EVT.

In addition, in the subgroup analyses, we revealed a similar direction and size for the effect of post-EVT SBP_max_ >165 mm Hg on the development of MCE. However, the adjusted OR were not significant for patients with successful recanalization, without history of hypertension, without intravenous antihypertensive agent and ICA occlusion. A possible explanation is that patients with successful recanalization have a lower rate of MCE [14% (51/364) vs. 34.3% (46/134)]. Additionally, the lack of association in patients without a history of hypertension or intravenous antihypertensive agents may be due to having a normal BP after EVT in these patients. Collateral circulation may be the main impact factor for the insignificant effect of BP on MCE in patients with ICA occlusion.

Notably, in addition to the mean SBP and SBP_max_, we also found that BP variability (SBP_SD_) was associated with the development of MCE. This result were in line with the findings of Skalidi et al.' study (24), which showed that increased values of the 24-h time rate of systolic BP variation are independently associated with formation of edema in acute stroke patients. Additionally, our study showed that the best SBP_max_ threshold that predicted the development of MCE was 165 mmHg, however, the difference in mean SBP (128 vs. 123 mm Hg) between the groups seems to be rather trivial. We speculate that the main reason for this phenomenon may be that the BPV is larger in the MCE group. These preliminary findings implied that in order to improve the outcomes of EVT patients, it is necessary to not only specify appropriate BP targets but also reduce BPV.

There are some limitations to this study. First, this was a retrospective study with a modest sample size, and we did not unify the protocol for post-thrombectomy BP management. Hence, the results should be interpreted with caution. Second, intraoperative BP is also an important parameter affecting the clinical outcome after EVT. Unfortunately, the intraprocedural BP was not analyzed. Third, we did not exclude patients with parenchymal hemorrhage, which may affect the assessment of MCE. However, such patients are rare. To our knowledge, we are the first group to investigate the association of post-procedural BP with the course of MCE in patients treated with EVT. Our study further expanded our understanding of the management of BP in patients with EVT.

In conclusion, higher mean SBP during the first 24 h after EVT is associated with the development of MCE in LVOS patients. Having an SBP_max_ >165 mm Hg was prospectively identified to best discriminate the development of MCE. Moreover, increasing BPV may pose a higher risk of developing MCE. These findings suggest that continuous BP monitoring after EVT could be used as a non-invasive predictor for clinical deterioration due to MCE. Randomized clinical studies are warranted to address BP goal after thrombectomy.

## Data Availability Statement

The raw data supporting the conclusions of this article will be made available by the authors, without undue reservation.

## Ethics Statement

The study was approved by the Ethics Committee of the First Affiliated Hospital of Wannan Medical College (201900039). The patients/participants provided their written informed consent to participate in this study.

## Author Contributions

All authors have contributed to the theoretical formalism, designing the study, data collection, data analysis, and writing the manuscript.

## Funding

This work was supported by the National Natural Science Foundation of China (Nos. 81870946 and 81530038), National Key Research and Development Program (No. 2017YFC1307901), and the Fundamental Research Funds for the Central Universities (No. WK9110000056).

## Conflict of Interest

The authors declare that the research was conducted in the absence of any commercial or financial relationships that could be construed as a potential conflict of interest.

## Publisher's Note

All claims expressed in this article are solely those of the authors and do not necessarily represent those of their affiliated organizations, or those of the publisher, the editors and the reviewers. Any product that may be evaluated in this article, or claim that may be made by its manufacturer, is not guaranteed or endorsed by the publisher.
